# Geometrical and electro-static determinants of protein-protein interactions

**DOI:** 10.6026/97320630017851

**Published:** 2021-10-31

**Authors:** Vicky Kumar, Ashita Sood, Anjana Munshi, Tarkeshwar Gautam, Mahesh Kulharia

**Affiliations:** 1 Centre for Computational Biology and Bioinformatics, School of Life Sciences, Central University of Himachal Pradesh, Kangra, India, 176206; 2 Department of Computational Sciences, School of Basic and Applied Sciences, CentralUniversity of Punjab, Bathinda, India, 151001; 3 Department of Human Genetics and Molecular Medicine, School of Health Sciences, Central University of Punjab, Bathinda, India, 151001; 4 Department of Zoology, Kalindi College, University of Delhi , Delhi, India

**Keywords:** Protein-protein interactions, protein-protein interaction interface, non-redundant database, residue interface propensity, hydrophobicity, solvation free energy, planarity, protrusion, depth

## Abstract

Protein-protein interactions (PPI) are pivotal to the numerous processes in the cell. Therefore, it is of interest to document the analysis of these interactions in terms of binding sites, topology of the interacting structures and physiochemical properties of interacting interfaces and the of forces interactions. The interaction interface of obligatory protein-protein complexes differs from that of the transient interactions. We have created a large database of protein-protein interactions containing over100 thousand interfaces. The structural redundancy was eliminated to obtain a non-redundant database of over 2,265 interaction interfaces. Therefore, it is of interest to document the analysis of these interactions in terms of binding sites, topology of the interacting structures and physiochemical properties of interacting interfaces and the offorces interactions. The residue interaction propensity and all of the rest of the parametric scores converged to a statistical indistinguishable common sub-range and followed the similar distribution trends for all three classes of sequence-based classifications PPInS. This indicates that the principles of molecular recognition are dependent on the preciseness of the fit in the interaction interfaces. Thus, it reinforces the importance of geometrical and electrostatic complementarity as the main determinants for PPIs.

## Background:

The cellular milieu where proteins perform their function is crowded. However, the spatial and temporal preciseness of interactions is rarely violated. This specificity and accuracy of the interacting proteins determines the fate of cells [1-3]. Protein-protein interactions (PPIs) form the very basis for all biological processes, such as signal transduction, material /energy transport, metabolic reactions, regulation of gene expression, cellular growth and proliferation[3-6]. These interactions form the fundamentals of the intracellular / intercellular communications [7]. Proteins therefore act as communication cogs, transferring the information through conformational changes and triggering corresponding transient structural adjustments in other molecules [8-9]. Understanding the protein interactions and enumerating the precise rules that govern these interactions mediate these molecular communication events can provide us with the deeper insights about metabolic and signaling network dynamics [10]. Any deviation from the normal interaction behavior of a protein leads to the gain or loss of a function, often leading to debilitating diseases such as neuro-degenerative diseases, cancer or even auto-immune problems [11].

The PPI manifests in formation of various types of complexes (viz. protein-protein complex, protein-DNA complex, protein-RNA complex, protein-membrane complex, protein-lipid complex, protein-carbohydrate complex, and others). In protein-protein complexes, the interaction of two or more proteins is very specific and is usually characterized by only a small subset of their surfaces [12-15]. Only those sites which own the proper binding features participate in the formation of PPCs [16-20] (Supplementary Figure 1 - see PDF). Such understanding may augment the development of computational tools for PPI sites prediction [9], and drug discovery [20-22]. In this direction, various research groups have examined the protein binding sites with respect to their size, shape, evolutionary conservation, chemical and amino acid composition, change in solvent accessibility of amino acids, and other such parameters. For example, Jones and Thornton, derived a parameter to determine the planarity index of the protein-protein interfaces [20]. As per their study, the average value of the planarity is 3.5 ± 1.7 Å for homodimers and 2.8 ± 0.9 Å for heterocomplexes. Bogan and Thorn showed that evolutionary conserved residues (often termed as hot spot residues) are the major contributor to the binding energy of the interactions [26]. Ozdemir et al. (2018) showed that a slight disruption in conserved residues more often results in change in the binding affinity and specificity [27]. Lo Conte et al. (1999) described the extent of burial of protein surface, during complex formation. He identified thisto be in the range of 1600 (±400) Å^2^ of interaction site for majority of heteromeric protein complexes. Bahadur et al. (2004) reported the abundance of aliphatic and aromatic residues and deficit of charges residues (except for Arg) in homodimeric interfaces in conformation with previous studies [21, 28,29,30]. We have studied protein-protein interaction interfaces (PPIIs) inPPInS and NRDB [31]. On the basis of sequence similarity between the interacting protein chains thePPIIs from both the datasets were classified into low-sequence, moderate-sequence, and high-sequence similarity classes. All three classes of NRDB dataset were examined for six important parameters of: residue interface propensity, hydrophobic content, solvation energy, compactness of interacting residue's neighborhood, planarity, and depth index.

## Materials & Methods:

### Datasets of protein-protein interaction interfaces:

#### Protein-protein interaction sitesbase (PPInS):

It has the protein-protein complexes (PPCs)as reported in PDB with their structural classification based onSCOPe (version 2.06). It harbors over 32000 X-ray crystallized structures of PPCs with structural resolution better than 2.5 Å. The information about these PPCs is available in the form of atom contact pairs wherein two atoms belonging to two different protein chains of a PPC were considered to be in contact if the intervening distance between them was less than the sum of their van der Waals radii plus 1 Å as a tolerance factor. We utilized the entire PPInS and its non-redundant form (NRDB) database for our study.

#### Categorization of PPIIs based on the sequence similarity in the interacting protein chains:

To study the influence of homo or heterodimeric nature of the proteins in PPIs, sequence similarity between the protein chains involved in the PPII was calculated using BLAST [32]. Based on sequence similarity observed, PPIIs were categorized into three classes. If the interacting protein chains were similar homologous up to 49%, then the corresponding PPII was marked under low sequence similarity (LSS) class; the PPIIs with protein chains sharing 50-89% sequence similarity were grouped under moderate sequence similarity (MSS) class; and the PPIIs with protein chains sharing 90-100% sequence similarity were grouped under high sequence similarity (HSS) class. All PPIIs from both of the datasets were categorized into three PPII classes.

### Calculation of residues' interface propensity:

Not all the amino acid residues favor their occurrence on the protein surface, some prefer to stay in the protein core thereby avoid or does not contribute much in protein complexation [28,33- 34]. The relative contribution of amino acids in promoting a protein site as the binding site is described as residue interface propensity (RIP) and is defined as the ratio of residue's relative contribution to the protein binding site to its relative contribution to the complete protein surface [35]. To calculate the RIP, PPIIs from the NRDB only were taken into consideration. The area contributed by an amino acid i to the protein binding site was calculated as the difference between its solvent accessible surface area (SASA) bearing its unbound and bound states. The propensity of a residue i to occur on the protein binding site (see PDF) was calculated using Eq 1 (see PDF).

### Analysis of protein-protein interaction interfaces/patches with respect to binding site parameters:

The binding nature of proteins is determined from physicochemical, structural, and evolutionary properties of their constituents favouring the non-covalent interactions with partner protein(s). Such properties bring two molecules closer, influence them for biological interactions, and define the destiny of the PPCs. Knowing the implications of protein binding sites analysis in protein engineering, all of the 223,714 PPIPs from PPInS and 4,530 PPIPs from NRDB were examined with respect to various PPI site parameters. While analyzing a PPIP, if an atom from a PPIP was seen interacting with more than one atom of the partner PPIP, its contribution in PPII formation was considered only for once.

### Hydrophobicity:

The hydrophobic residues are reported in abundance of PPI sites [36]. The kinetics of interfaces with predominant hydrophobic residues are reported to be different than hydrophilic ones because the tightly bound aquasphere of surface bound water molecules acts as an additional barrier that has to be removed before direct protein-protein interaction can take place[29]. To determine the level of hydrophobicity (Φ) associated with PPIPs, the hydrophobicity scale for amino acids was used from literature[37]. For each interacting atom in the PPIP, its corresponding hydrophobicity score was obtained by dividing the residue hydrophobicity score by the number of atoms of the residue. The hydrophobicity score of all interacting atoms in the protein-protein interaction patch (PPIP) was calculated by linear augmentation to represent the hydrophobicity score of the PPIP (Eq. 2 and 3 - see PDF). 

### Solvation free energy:

The solvation free energy of amino acids from the interacting protein also influences its kinetics, hence we calculated the solvation free energy (ω) of PPIP by linear summation of the average individual contributions of the interacting atoms (Eq. 4 and 5 - see PDF). The solvation free energy scale for amino acids given by Wimley et al., 1996 [38] was used. (see PDF)

### Size of the interacting patch:

The protein-protein interaction patches are generally very small region on the protein surface withvery specificcertain structural / thermodynamical features. We determined the size of such interaction interfaces by summing up the difference of the solvent accessible surface area (SASA) of the atoms in the bound and unbound forms. (see PDF)

### Depth index:

Contrary to the ASA,the depth index of an amino acid indicates the extent to which an amino acid is buried in the protein core. The location of amino acids in proteins is determined using their solvent accessibility. An amino acid is said to exist on protein surface if the sum of solvent accessibilities for all of its constituting atoms is a non-zero value. While the amino acids with zero solvent accessibility are considered to be buried in the protein core [39]. The depth index of PPIP (ζ) was computed using PSAIA [30]. The depth index of each interacting atom from a PPIP was summed up and represented as a depth index of the PPIP (Eq. 7) (see PDF)

### Protrusion index:

The protrusion index studies the topology of the interface site and gives the measure of how much dense is the neighborhood of an atom on the protein surface [40].The protrusion index of PPIPs Ψ) was also determined by using PSAIA [30] in a manner similar to the depth index for all non-hydrogen atoms (Eq. 8). (see PDF)

### Planarity index:

The protein binding sites are flat and circular in shape [41]. The calculation of root mean square deviation of all the surface atoms from the least-squares plane (derived from the surface atoms) gives the planarity index of the interacting interfaces. If all atoms correctly fit a plane, the planarity index comes out to be zero.To calculate the planarity index of the PPIPs, *princip* function of the SURFNET [42] was used. Using *princip*, an equation of plane was derived by employing the coordinates of the interacting atoms in the PPIP. Following this, the root-mean-square deviation (RMSD) of interacting atoms from the derived plane was determined and designated as the planarity index of the PPIPs.

### Statistical analysis of PPII parametric scores obtained from PPInS and NRDB analysis:

The overall trends of parameteric score distribution wereapparently very similar. Hence, the statistical aspect of the data was explored. The distributions of PPIP parametric scores (after removal of 1% statistical outliers) for each PPI site parameter from all three PPII classes were taken into the consideration. For each PPI site parameter, the mean and standard deviation of parametric scores were calculated with respect to each PPII class separately. Thereafter, for each PPII class, p-value describing the statistical significance between the parametric score of three PPII classes was calculated using two-tailed ANOVA test.

## Results and Discussion:

We examined the PPIPs derived from experimentally determined PPCs in terms of various physicochemical and geometrical properties. Two datasets viz. PPInS and NRDB where interaction sites were demarcated based on the inter atomic distance between the constituents of two protein chains of the PPCs, were considered for the study. The collection of ACPs between two protein chains of a PPC were referred as the PPII while the collection of atoms involved in PPII from each interacting protein chains were termed as the PPIPs. All the PPIIs from PPInS and NRDB were categorized into three separate classes viz. LSS, MSS, and HSS by looking at the sequence similarity between the protein chains involved in formation of PPII under consideration (Table 1 - see PDF). Out of total 111,857 PPIIs in PPInS, around 73% of PPIIs were formed by the protein chains sharing HSS. Around 25% PPIIs were formed by the protein chains sharing LSS, and only 2% PPIIs were results of the interaction between protein chains sharing MSS. For NRDB, the values for PPIIs with HSS, LSS, and MSS were around 62%, 32%, and 6%, respectively. This showed the presence of homodimers in abundance. The possible reasons for this may be the fact that the origin of life started with interactions in absolutely homologous proteins. However, through the course of evolution, perturbation in the genomic code might have caused the formation of heterologous protein complexes. Similar findings have been reported by (Winter el al., 2002) [43]. The factors which might have played a crucial role in bringing two heterologous protein units closer must be their physicochemical, geometrical and other characteristics.

## Calculation of residue interface propensity (RIP):

The RIP was calculated separately for all three classes of PPIIs (LSS, MSS, and HSS) of NRDB (Figure 1 - see PDF). The propensity scores that we have obtained are quite similar to those which were earlier proposed [21-22]. The higher propensity for aromatic amino acids (Tyr, Phe, and Trp) and aliphatic hydrophobic amino acids (Met, Cys, Ile, Leu, Val) on interacting interface is reported in these studies unanimously. In other studies [26,39, 44] too, the aromatic residues were reported in abundance on interaction sites. One reason behind this greater occurrence is the predominant contribution of solvation and hydrophobic effect [45,46]. The small amino acids such as Ala, Gly, Ser, and Thr is comparatively marginal, they have no specific tendency to either avoid or favour the PPIIs in terms of occurrence. Asn, Gln, and Pro too are borderline PPII avoiders while His slightly favours its occurrence on the PPIIs. It is necessary to point out that His exists in multiple protonation states and our data does not differentiate among these. It is possible that some protonation states could decisively dis/favour the PPIIs, and our data is only an average of the overall effect. The charged amino acids (Lys, Glu, Asp), with the exception of Arg (which was relatively neutral), had the least propensity to occur on PPIIs and this was also reported by many groups [21,22]. The reduced presence of Glu and Asp on PPIIs is perhaps rooted in their inability to form interaction with aromatic hydrophobic amino acids. Although Lys has the ability to form cation-Pi interaction, yet the conformational entropy associated with multiple single bonds would not favour the PPI.

## Analysis of PPIPs from PPInS and NRDB:

The result obtained by analyzing the PPIPs from all the three PPII classes (LSS, MSS, and HSS) of the PPInS and NRDB dataset are given in Table 3 and 4(see PDF), respectively. The parametric scores for all the PPIPs from LSS, MSS, and HSS are shown graphically in supplementary figures S2-S7 (see PDF)enmasse. In Table 5(see PDF), the statistical analysis of PPIP parametric scores is presented in terms of mean, standard deviation, and p-value (calculated using two-tail ANOVA test). The statistical analysis was carried out considering all three PPII classes of both the datasets separately as well as collectively.

## Analysis of PPIPs from the PPInS dataset:

The PPIPs were analyzed with respect to hydrophobicity, solvation free energy, depth index, size of the interacting patch, protrusion index, and planarity. It is pertinent to mention that on analyzing PPIPs from PPInS, initially, a significant difference between the parametric scores (obtained with respect to each PPI site parameter) of the three PPII classes was observed (Table 3 - see PDF). However, on removing less than 1% PPIPs (statistical outliers) from each PPII class, the cumulative parametric scores for depth, protrusion, and planarity index of PPIPs from all three classes of PPII reduced down to the identical ranges. Similarly, the cumulative score for solvation free energy and hydrophobic content of PPIPs also were also seen converging to a common sub-range. The possibility that these trends were on account of proportional redundancies of PDB, we looked for the same patterns in the NRDB.

## Analysis of PPIPs from NRDB dataset:

The analysis of PPIPs from NRDB considering both the cases, i.e. analysis of all PPIP from each PPII class and analysis of PPIPs after removing less than 1% statistical outlier from each PPII class, also showed the similar trends for the parametric scores for each PPI site parameter (Table 4 - see PDF). Here, the range of parametric scores for each PPIP parameter (except for the planarity) showed the greater variability. After removal of outliers, only the PPIPs from LSS and MSS class found to converge into a common sub-range for parametric scores. However, this could be explained by the methodology of database creation. At the time of NRDB creation, for each pair of SCOP superfamily pair, the PPII selected as a part of NRDB was the one with maximum number of ACPs among all the PPIIs sharing the same SCOP superfamily pair. Therefore, it was obvious for the larger PPIPs to possess relatively higher cumulative hydrophobicity, solvation energy, and other parametric scores. But, as the planarity index of PPIPs is not much dependent on the size of interacting patches (i.e. PPIPs), the cumulative parametric score obtained for planarity for each PPII class, confined to a common sub-range.

The size of PPIPs varied for each of these classes (MSS, LSS, and HSS) and it was least for MSS and highest for the HSS. The maximal tail size of the interacting patch of LSS was almost 1300 Å^2^ less than HSS and 1300 Å^2^ more than MSS. The effect of size can be seen for the rest of the parameters except for planarity index. Planarity score was an average measure of RMSD of PPIP constituent atoms from the best-fit plane, hence this value was almost size independent. The hydrophobicity showed the largest variation, with range for values corresponding to HSS (-1 - 93) was almost 3 times that of LSS (-1 - 36) and MSS (0 - 31). The parametric range for cumulative depth and protrusion index for HSS (Depth: 0 - 28 and Protrusion: 0 - 150) was almost twice that of the other two classes. The upper and lower meniscus for solvation free energy of HSS (-29.0 kJ·mol-1- 32 kJ·mol-1) was very broad as that of LSS (-6.0 kJ·mol-1 - 21 kJ·mol-1) and MSS (-5.0 kJ·mol-1- 23 kJ·mol-1). In Supplementary Figures 8-13(see PDF), the trend of parametric values obtained for around 99% of the PPIPs from each of the three PPII classes of both PPInS and NRDB datasets with respect to six PPI parameters are shown.

## Analysis of PPIPs with respect to the hydrophobicity:

The hydrophobic analysis of PPIPs from PPInS and NRDB revealed that even though the range of hydrophobicity values of PPIPs differed among HSS, MSS, and LSS (Figure 2 - see PDF), the distribution of the parametric scores followed almost the same pattern. The statistical analysis of parametric score distribution with respect to mean, standard deviation, and p-values (Table 5 - see PDF) support the hypothesis that the cumulative hydrophobic content of PPIP from all the PPII classes is significantly same. And, this was applicable for PPIIs from both the datasets. The hydrophobicity score for the PPInS is multi-peak, whereas the same obtained from NRDB is single-peak. This apparent difference is perhaps due to the high order of redundancy in PDB for PPI complexes with the relatively larger contribution of hydrophobic effect in the binding energy. As the hydrophobicity is a major contributor to the formation of protein crystals [47,50], thus, the majority of the PPInS have a very high hydrophobicity component in the interacting energetics. This redundancy is removed in the NRDB and as a consequence of which the multi-peak distribution converges to a single-peak. Clearly, the hydrophobicity values for PPIPs can vary from as low as 100 to as high as 0 (NRDB part of Figure 3 - see PDF) (See methods section). However, the majority of the PPIPs have the hydrophobic score of 5-8 irrespective of homo/heterodimeric nature of complexes. The analysis made by [49-50] concluded that the hydrophobic effect is predominated contributor in the formation of obligate complexes. The trends of the cumulative hydrophobic index of the PPIPs as seen in Figure 2 (see PDF), clearly shows that the proportionate-contribution of hydrophobic effect (on an average) is independent of the extent of sequence similarity between the interacting interfaces.

## Analysis of PPIPs with respect to solvation free energy (in KJ/Mol):

The range of solvation free energy values of PPIP does not differ amongst HSS, MSS, and LSS (Figure 3 - see PDF) as the graphs follow an identical pattern with statistical similarity among the parametric scores of different PPII classes (Table 5 - see PDF). This was true for NRDB too. This similarity is perhaps due to the uniform nature of PPI, which is also one major reason that solvation-energy based prediction tools for PPI and drugability studies are more successful [51-52]. Interestingly the nature of complexes - whether homodimeric or heterodimeric doesn't influence the peak of solvation free energies in NRDB or PPInS.

## Analysis of PPIPs with respect to the depth index:

The distribution range of the depth index of PPIP also does not differ amongst the three classes of PPInS and NRDB both, as seen in Figure 4(see PDF) where the graphs follow an identical pattern. The depth index of the PPInS is the same as obtained for the PPIPs from NRDB with single-peak with similar shoulders. The statistical analysis of cumulative depth distribution for each PPII class of the both of these datasets was also significantly same (Table 5 - see PDF). The very low value of cumulative depth index shows that the atoms involved in PPIPs are on the surface. This may be due to the effect of interacting surface induction. The majority of PPIPs have identical cumulative depth index and this fact is not influenced by the extent of redundancy in the datasets. Irrespective of the structural/functional class of the protein, the cumulative depth index of the PPIP remains more or less constant. This is another indication that irrespective of the homo/heterodimers, the physicochemical and structural parameters governing PPI occupy the same value-space.

## Analysis of PPIPs with respect to the size of interacting patch (in Å^2^):

The size of interacting patches has a direct relationship with the van der Waals (vdW) forces of interactions. Greater the size of PPIP, larger is the vdW interaction energy of PPIPs. In earlier studies [18,53], a limited number of PPCs were studied and it was observed that on an average PPI had a size of 800 ± 400 Å^2^. This is similar to earlier reportswhich reported the size of the interfaces as small as ~800 Å^2^[19]. In another study [53] the size of interfaces was found in the range of 415 to 3568 Å^2^ for heterodimers, 550 to 4718 Å^2^ for homodimers, and 423 to 2361 Å^2^ for transient complexes. In our study too, the interfaces from PPInS dataset are reported with size upto 6500 Å^2^ (Figure 5-Left - see PDF)) while in NRDB interaction interfaces are reported to be between 112 Å^2^ to 8400 Å^2^ (Figure 5-Right - see PDF)per interacting partner. However, most of the PPIIs from PPInS and NRDB were seen covering protein surface up to within 800 ± 400 Å^2^ and 1200 Å^2^ ± 400 Å^2^,respectively in conformity with earlier studies [21,22]. In Figure 6(see PDF), the interacting region of protein chains with smallest as well as largest PPIP size from NRDB and PPInS is shown. For this parameter too, the statistical analysis carried out to analyze the distribution of PPIP size, considering all PPII classes of both the datasets, showed the similarity in terms of the PPIP size (Table 5 - see PDF).

## Analysis of PPIPs with respect to the protrusion index:

The protrusion index or the compactness of neighborhood of interacting residues has been studied by some groups [56] and it has been seen that its average value ranges from 0 to 14 [40] for protein atoms. In our datasets, we studied the cumulative protrusion index (Figure 7 - see PDF). Surprisingly its value (between 7-10) was very low, considering the large number of atoms that contribute to the PPII. This indicates the relatively higher packing (thus increased neighbor density) and perhaps also reduced flexibility as reported earlier [56]. This is an important parameter for prediction of hot-spots residues on PPI sites. Irrespective of sequence similarity between the interacting partners protrusion index followed an identical distribution (Table 5 - see PDF). Interestingly the nature of complexes – whether homodimeric or heterodimeric does not differentially influence the protrusion index (compact packaging of the PPI site).

##  Analysis of PPIPs with respect to the planarity index

The planarity analysis of PPIPs revealed the clear independency of the type and size of protein in terms of their binding site preferences (Figure 8 - see PDF). For both the datasets, PPInS (which is repository of over two hundred thousand PPIPs) and the NRDB (having PPIPs with size up to 8400 Å^2^), the deviation of interacting atoms from the derived plane was found in the range of 0-8 Å. Maximum number of PPIPs were reported with deviation of 1-5 Å which is analogous to the previous findings [19,28,51,53,57]. These findings hold true for all three respective PPII classes of both the datasets. The analysis of parametric score distributions with respect to three PPII classes of the both the datasets also showed the similarity in terms of their level of flatness (Table 5 - see PDF).

## Conclusion:

The protein-protein interaction interfaces from two datasets, covering the largest collection of experimentally determined protein-protein complexes ever, were examined in terms of their hydrophobic content, associated solvation energy, compactness of interacting residue' neighborhood, planarity and depth index. Analysis of PPIPs from NRDB pertaining to RIP showed the presence of aliphatic and aromatic residues on interaction sites in abundance and deficit of charged residues (except Arg) as reported in previous studies. On analyzing PPIPs from PPInS, initially, a significant difference between the parametric scores (obtained with respect to each PPI site parameter) of the three PPII classes was observed. However, on removing less than 1% PPIPs (statistical outliers) from each PPII class, the cumulative parametric scores for each PPI site parameter from all three classes of PPII reduced down to the identical ranges. The analysis of PPIPs from NRDB considering each PPII class (with and without outliers) also showed the similar trends for the parametric scores for each PPI site parameter, however, with greater variability (except for the planarity). As the PPIPs in HSS class were relatively larger in size, the resulting cumulative score could not get aligned with the scores from LSS and MSS wherein they were found to converge into a common sub-range after outlier removal. However, overall, the objective analysis of PPIPs (from all three PPII classes of both the datasets) with respect to all PPI sites parameters showed the similar trends. This indicates that the principles of molecular recognition among proteins are not driven by their sequence / structural similarity and reinforces the importance of geometrical and electrostatic complementarity as the main component for PPIs.

## Author contribution:

The proposed work was conceptualized by MK and executed by VK and MK. The data was analyzed by VK, AM, TG and MK. The manuscript was written by VK, AS and MK. All authors have read and approved the manuscript prior to submission.

## Abbreviations:

PPInS - Protein-protein interaction sitesbase

NRDB - Non-redundant database

ACP - Atomic contact pair

PPII - Protein-protein interaction interface

PPIP - Protein-protein interacting patch

RIP - Residue interface propensity

LSS - Low sequence similarity

MSS - Moderate sequence similarity

HSS - High sequence similarity

PDB - Protein Data Bank

PPC - Protein-protein complex
